# Retention in HIV Primary Care Using a Web-Based Patient Engagement Platform: Multistate Case-Control Study

**DOI:** 10.2196/64092

**Published:** 2024-10-02

**Authors:** Adam Carl Sukhija-Cohen, Henna Patani, Michael Foxworth Blasingame, Kathy Linh Vu, Ramin Bastani

**Affiliations:** 1 Sutter Health Palo Alto Medical Foundation Research Institute Palo Alto, CA United States; 2 Public Health Division AIDS Healthcare Foundation Los Angeles, CA United States; 3 Healthvana Los Angeles, CA United States; 4 Research Triangle Institute Research Triangle Park, CA United States

**Keywords:** HIV, primary health care, retention in care, digital technology, appointments and schedules

## Abstract

**Background:**

Digital interventions to improve retention in HIV care are critical to ensure viral suppression and prevent further transmission. AIDS Healthcare Foundation Healthcare Centers are centers across the United States that provide primary HIV care. Traditionally, the Healthcare Centers conduct phone calls with patients to schedule and confirm appointments, as well as share laboratory results. In 2017, Healthvana piloted a digital platform at AIDS Healthcare Foundation Healthcare Centers to send patients SMS text message appointment reminders and allow patients to review their upcoming appointment and view their laboratory results in the web-based patient portal.

**Objective:**

A national implementation in 15 US states and Washington, DC, of this digital intervention pilot by Healthvana aims to determine whether SMS appointment reminders and web-based patient portal logins improved retention in care compared to traditional methods.

**Methods:**

A retrospective analysis of 40,028 patients living with HIV was conducted at the 61 AIDS Healthcare Foundation Healthcare Centers between January 2, 2017, and May 22, 2018. Patients were invited to enroll in Healthvana’s digital intervention pilot, allowing for a natural, organization-wide case-control study. Separate binary logistic regression models evaluated the relationship between receiving SMS appointment reminders and completing scheduled appointments, as well as the relationship between logging into the web-based patient portal and completing scheduled appointments. Four scheduled consecutive appointments for each patient were included in the analysis to account for 1 full year of data per patient.

**Results:**

Patients who received the SMS appointment reminder were 1.7 times more likely to complete appointment 1 compared to patients who did not receive the SMS appointment reminder (*P*<.001). In addition, patients who received the SMS appointment reminder were 1.6 times more likely to complete appointment 2 (*P*<.001), 1.7 times more likely to complete appointment 3 (*P*<.001), and 1.8 times more likely to complete appointment 4 (*P*<.001) compared to patients who did not receive the SMS appointment reminder. Patients who logged in to the web-based patient portal prior to their scheduled appointment were 7.4 times more likely to complete appointment 1 compared to patients who did not log in (*P*<.001). In addition, patients who logged in to the web-based patient portal prior to their scheduled appointment were 3.6 times more likely to complete appointment 2 (*P*<.001), 3.2 times more likely to complete appointment 3 (*P*<.001), and 2.8 times more likely to complete appointment 4 (*P*<.001) compared to patients who did not log in.

**Conclusions:**

HIV primary care appointment completion was higher when patients engaged with Healthvana’s digital platform. Digital technology interventions to ensure patients complete their scheduled HIV care appointments are imperative to curb the HIV epidemic.

## Introduction

There are 1.2 million people living with HIV in the United States [1]. A public health model called the HIV care continuum represents 4 important stages of HIV care [2]. Across the continuum, 87% of people living with HIV are diagnosed, 66% have received HIV medical care, 50% are retained in care, and 57% have achieved viral suppression.

People living with HIV retained in care—that is, those who have continuously scheduled medical care, defined as having 2 or more cluster of differentiation 4 (CD4) or viral load tests at least 3 months apart—are at the lowest rate among the 4 steps [1]. People living with HIV who are aware of their HIV status and are either not in care or not virally suppressed account for nearly two-thirds of new infections [3]. Therefore, implementing innovative interventions is important to retain people living with HIV in care, increasing their likelihood of achieving viral suppression and preventing further transmissions [2-5].

AIDS Healthcare Foundation (AHF) provides HIV primary care at 61 AHF Healthcare Centers in 15 states (California, Florida, Georgia, Illinois, Indiana, Louisiana, Maryland, Mississippi, Nevada, New York, Ohio, Pennsylvania, South Carolina, Texas, and Washington) and Washington, DC. These are all priority states with a disproportionate burden of new HIV diagnoses according to the Ending the HIV Epidemic in the US initiative [6]. The centers operate in major metropolitan areas and offer standardized care.

Traditionally, AHF Healthcare Centers call patients to schedule and confirm appointments and share laboratory results. Beginning in 2017, an AHF/Healthvana collaboration pilot-tested a digital platform that sends patients SMS text message appointment reminders and allows patients to access a web-based patient portal. Within the portal, Healthvana features customized laboratory result delivery, 2-way communication between patients and health care providers, and appointment management. This type of digital intervention has been shown to improve HIV care across the care continuum [7-11]. However, few studies have used large sample sizes to evaluate the effect of digital intervention on HIV care retention. This pilot study aims to determine whether SMS appointment reminders and patient portal logins improved retention in care nationally compared to traditional methods.

## Methods

A retrospective analysis of all AHF patients was conducted at the 61 AHF Healthcare Centers between January 2, 2017, and May 22, 2018. AHF recommendations call for patients to complete an appointment every 3 months. Therefore, 4 scheduled consecutive appointments for each patient are included in the analysis to account for 1 full year of data per patient.

Patients were invited to enroll in Healthvana’s digital intervention pilot test, allowing for a natural, organization-wide case-control study. Demographics retrospectively extracted from the entire patient population included age group, gender, race, and health insurance type. The 2 predictor variables were SMS appointment reminders and web-based patient portal login. Patients in the “SMS appointment reminder” category were those who affirmed receiving an SMS reminder before their scheduled appointment. Only patients who consented to receiving SMS reminders received them; those who did not consent or opted out had SMS reminders removed entirely. Patients in the “web-based patient portal login” category were those who logged in to the portal before their scheduled appointment. As part of AHF Healthcare Center services, all patients create a username and password for Healthvana’s patient portal; however, patients are not required to log in as part of their appointment. The outcome variable, “scheduled appointment,” is categorized as “completed” versus “not completed.” Logistic regression models were built to determine the odds of patients completing their appointments.

### Ethical Considerations

Healthvana securely collects, stores, and maintains AHF health data in encrypted format both in transit and at rest and is Health Insurance Portability and Accountability Act–compliant. For this evaluation, data were extracted and deidentified from Healthvana’s digital platform by Healthvana staff. All analyses were conducted by the AHF research team in SAS (version 9.4; SAS Institute). As a quality improvement pilot study requested by AHF leadership, this retrospective analysis received exemption from the Western Institutional Review Board.

## Results

### Overview

Table 1 depicts demographic characteristics. Most patients scheduled appointment 1 (n=40,028), followed by appointment 2 (n=37,878), appointment 3 (n=34,866), and appointment 4 (n=31,550). Most patients in appointment 1 were aged 50-54 (n=5418, 13.5%) or 25-29 years (n=5226, 13.1%), male (n=32,756, 81.8%), and White (n=21,633, 54%) or Black/African American (n=17,361, 43.4%). Most patients had private insurance (n=22,401, 56%), followed by insurance provided by the Ryan White HIV/AIDS Program (n=9121, 22.8%) or Medicare/Medicaid (n=8506, 21.3%). Distributions of age group, gender, and race did not change significantly across the 4 appointments (*χ*^2^ distribution tests; *P*>.05 for all groups). However, insurance type distribution differed significantly across the 4 appointments: private insurance decreased, and Medicare/Medicaid and Ryan White increased over time (*χ*^2^ distribution test; *P*<.001).

Table 2 shows appointment outcomes based on SMS reminders before scheduled appointments. A total of 1.7% (664/40,028) of patients received reminders for appointment 1, 13.3% (5037/37,878) for appointment 2, 22.1% (7698/34,866) for appointment 3, and 28.3% (8944/31,550) for appointment 4.

Table 3 describes the patients’ appointment outcomes based on whether they logged in to the web-based patient portal; 19.2% (7705/40,028) logged in for appointment 1, 22.7% (8591/37,878) for appointment 2, 25.6% (8913/34,866) for appointment 3, and 26.8% (8469/31,550) for appointment 4. Overall, 71.3% (28,540/40,028) of patients who scheduled appointment 1 completed the appointment. A total of 71.1% (28,004/39,364) of patients who did not receive the SMS appointment reminder completed appointment 1; in contrast, 80.7% (536/664) of patients who received the SMS reminder completed appointment 1. By patient portal, 66% (21,338/32,323) of patients who did not log in to the patient portal completed appointment 1; on the other hand, 93.5% (7202/7,705) of patients who logged in to the patient portal prior to their scheduled appointment completed appointment 1. For each subsequent scheduled appointment, patients who either received the SMS appointment reminder or who logged in to the patient portal prior to their scheduled appointment had a higher proportion of scheduled appointment completions compared to patients who either did not receive the SMS appointment reminder or did not log in to the patient portal.

In separate logistic regression models (Table 4), patients who received the SMS appointment reminder before their scheduled appointment were 1.7 times more likely to complete appointment 1 compared to patients who did not receive the reminder (odds ratio [OR] 1.7, 95% CI 1.4-2.1; *P*<.001); the likelihood increased 1.6 times for appointment 2 (OR 1.6, 95% CI 1.5-1.7; *P*<.001), 1.7 times for appointment 3 (OR 1.7, 95% CI 1.6-1.8; *P*<.001), and 1.8 times for appointment 4 (OR 1.8, 95% CI 1.6-1.8; *P*<.001). Patients who logged in to the patient portal before their scheduled appointment were 7.4 times more likely to complete appointment 1 compared to patients who did not log in (OR 7.4, 95% CI 6.7-8.1; *P*<.001); the likelihood increased 3.6 times for appointment 2 (OR 3.6, 95% CI 3.4-3.8; *P*<.001), 3.2 times for appointment 3 (OR 3.2, 95% CI 3.0-3.4; *P*<.001), and 2.8 times for appointment 4 (OR 2.8, 95% CI 2.7-3.0; *P*<.001).

**Table 1:** AIDS Healthcare Foundation (AHF) patient demographics by scheduled AHF Healthcare Center appointment, January 2, 2017, to May 22, 2018.

			Appointment 1 (n=40,028), n (%)		Appointment 2 (n=37,878), n (%)		Appointment 3 (n=34,866), n (%)		Appointment 4 (n=31,550), n (%)
**Age group (years)**									
	18-24	2407 (6)		2219 (5.9)		1956 (5.6)		1715 (5.4)	
	25-29	5226 (13.1)		4885 (12.9)		4405 (12.6)		3886 (12.3)	
	30-34	4993 (12.5)		4686 (12.4)		4296 (12.3)		3886 (12.3)	
	35-39	4415 (11)		4180 (11)		3822 (11)		3443 (10.9)	
	40-44	3929 (9.8)		3709 (9.8)		3436 (9.9)		3116 (9.9)	
	45-49	4614 (11.5)		4399 (11.6)		4093 (11.7)		3713 (11.8)	
	50-54	5418 (13.5)		5186 (13.7)		4843 (13.9)		4420 (14)	
	55-59	4254 (10.6)		4070 (10.7)		3810 (10.9)		3503 (11.1)	
	60-64	2606 (6.5)		2510 (6.6)		2328 (6.7)		2156 (6.8)	
	≥65	2166 (5.4)		2034 (5.4)		1877 (5.4)		1712 (5.4)	
**Gender**									
	Female	7228 (18.1)		6738 (17.8)		6177 (17.7)		5592 (17.7)	
	Male	32,756 (81.8)		31,096 (82.1)		28,646 (82.2)		25,917 (82.1)	
	Transgender	44 (0.1)		44 (0.1)		43 (0.1)		41 (0.1)	
**Race**									
	American Indian/Alaska Native	87 (0.2)		86 (0.2)		83 (0.2)		72 (0.2)	
	Asian	790 (2)		728 (1.9)		659 (1.9)		579 (1.8)	
	Black/African American	17,361 (43.4)		16,586 (43.8)		15,361 (44)		14,016 (44.4)	
	Multiracial	71 (0.2)		65 (0.2)		57 (0.2)		50 (0.2)	
	Native Hawaiian/Pacific Islander	86 (0.2)		83 (0.2)		80 (0.2)		72 (0.2)	
	White	21,633 (54)		20,330 (53.7)		18,626 (53.4)		16,761 (53.1)	
**Insurance type**									
	Medicare/Medicaid	8506 (21.3)		8189 (21.6)		7693 (22.1)		7090 (22.5)	
	Private insurance	22,401 (56)		20,889 (55.1)		18,973 (54.4)		16,973 (53.8)	
	Ryan White HIV/AIDS Program	9121 (22.8)		8800 (23.2)		8200 (23.5)		7487 (23.7)	

**Table 2:** Outcome of AIDS Healthcare Foundation Healthcare Center patient appointments by SMS appointment reminder and appointment number, January 2, 2017, to May 22, 2018.

Appointment			Did not receive the SMS appointment reminder, n/N (%)		Received the SMS appointment reminder, n/N (%)		Total, n/N (%)
**Appointment 1**							
	Completed	28,004/39,364 (71.1)		536/664 (80.7)		28,540/40,028 (71.3)	
	Not completed	11,360/39,364 (28.9)		128/664 (19.3)		11,488/40,028 (28.7)	
	Total	39,364/40,028 (98.3)		664/40,028 (1.7)		—^a^	
**Appointment 2**							
	Completed	20,510/32,841 (62.5)		3643/5037 (72.3)		24,153/37,878 (63.8)	
	Not completed	12,331/32,841 (37.5)		1394/5037 (27.7)		13,725/37,878 (36.2)	
	Total	32,841/37,878 (86.7)		5037/37,878 (13.3)		—	
**Appointment 3**							
	Completed	15,578/27,168 (57.3)		5355/7698 (69.6)		20,933/34,866 (60)	
	Not completed	11,590/27,168 (42.7)		2343/7698 (30.4)		13,933/34,866 (40)	
	Total	27,168/34,866 (77.9)		7698/34,866 (22.1)		—	
**Appointment 4**							
	Completed	12,317/22,606 (54.5)		6054/8944 (67.7)		18,371/31,550 (58.2)	
	Not completed	10,289/22,606 (45.5)		2890/8944 (32.3)		13,179/31,550 (41.8)	
	Total	22,606/31,550 (71.7)		8944/31,550 (28.3)		—	

^a^Not applicable.

**Table 3:** Outcome of AIDS Healthcare Foundation Healthcare Center patient appointments by web-based patient portal login and appointment number, January 2, 2017, to May 22, 2018.

Appointment			Did not log in to the web-based patient portal, n/N (%)		Logged in to the web-based patient portal, n/N (%)		Total, n/N (%)
**Appointment 1**							
	Completed	21,338/32,323 (66)		7202/7705 (93.5)		28,540/40,028 (71.3)	
	Not completed	10,985/32,323 (34)		503/7705 (6.5)		11,488/40,028 (28.7)	
	Total	32,323/40,028 (80.8)		7705/40,028 (19.2)		—^a^	
**Appointment 2**							
	Completed	17,004/29,287 (58.1)		7149/8591 (83.2)		24,153/37,878 (63.8)	
	Not completed	12,283/29,287 (41.9)		1442/8591 (16.8)		13,725/37,878 (36.2)	
	Total	29,287/37,878 (77.3)		8591/37,878 (22.7)		—	
**Appointment 3**							
	Completed	13,929/25,953 (53.7)		7004/8913 (78.6)		20,933/34,866 (60)	
	Not completed	12,024/25,953 (46.3)		1909/8913 (21.4)		13,933/34,866 (40)	
	Total	25,953/34,866 (74.4)		8913/34,866 (25.6)		—	
**Appointment 4**							
	Completed	11,993/23,081 (52)		6378/8469 (75.3)		18,371/31,550 (58.2)	
	Not completed	11,088/23,081 (48)		2091/8469 (24.7)		13,179/31,550 (41.8)	
	Total	23,081/31,550 (73.2)		8469/31,550 (26.8)		—	

^a^Not applicable.

**Table 4:** Odds ratios (ORs) for completing scheduled appointments at AIDS Healthcare Foundation Healthcare Centers by SMS appointment reminder, web-based patient portal login, and appointment number, January 2, 2017, to May 22, 2018.

Appointment completed	Received the SMS appointment reminder			Logged in to the web-based patient portal	
	OR (95% CI)	*P* value	OR (95% CI)		*P* value
Appointment 1	1.7 (1.4-2.1)	<.001	7.4 (6.7-8.1)		<.001
Appointment 2	1.6 (1.5-1.7)	<.001	3.6 (3.4-3.8)		<.001
Appointment 3	1.7 (1.6-1.8)	<.001	3.2 (3.0-3.4)		<.001
Appointment 4	1.8 (1.6-1.8)	<.001	2.8 (2.7-3.0)		<.001

## Discussion

Healthvana’s digital platform was associated with AHF patients completing their scheduled AHF Healthcare Center appointments. Patients who received the SMS appointment reminder and patients who logged into the web-based patient portal prior to their scheduled appointments were more likely to complete their scheduled appointments compared to patients who did not receive the SMS appointment reminder or did not log in to the patient portal. These findings suggest engaging patients in HIV care using a digital platform can help improve retention—a critical step in the HIV care continuum.

There are 2 strengths in this study. First, data across 15 states and Washington, DC, were analyzed, increasing generalizability and reducing geographic bias. Second, this study includes a very large sample size of patients, enabling us to rule out spurious associations. Within the current body of research, studies are often limited to 1 or 2 jurisdictions and have small sample sizes [7-11].

This study includes important limitations. Healthvana’s digital platform was pilot-tested at different time points during the study duration; this study did not distinguish whether patients had the option to engage with the digital platform. In addition, this study did not include socioeconomic factors that may affect an individual’s access to a mobile phone to receive SMS appointment reminders or a smartphone or computer to log in to the patient portal. Lastly, this study did not assess the reasons patients’ appointments were not completed.

Clinic-level barriers, including understaffing, can undermine HIV prevention and care at all stages of the HIV care continuum [12,13]. The capacity of staff to engage with people living with HIV in timely and consistent intervals is an important structural factor digital platforms can address. This study found significant improvements in scheduled appointment completion with a large patient population. Future HIV research should continue to focus on digital platforms that can improve retention in care and viral suppression across HIV care settings and insurance coverage (ie, federally qualified health centers and private primary care clinics).

## Abbreviations

AHF: AIDS Healthcare Foundation
CD4: cluster of differentiation 4
OR: odds ratio

## Data Availability

The data set analyzed during this study is not publicly available due to data protections for people living with HIV. However, the data are available on reasonable request.
